# Designing monitoring protocols to measure population trends of threatened insects: A case study of the cryptic, flightless grasshopper *Brachaspis robustus*

**DOI:** 10.1371/journal.pone.0238636

**Published:** 2020-09-24

**Authors:** Jennifer C. Schori, Tammy E. Steeves, Tara J. Murray

**Affiliations:** 1 School of Biological Sciences, College of Science, University of Canterbury, Christchurch, New Zealand; 2 School of Forestry, College of Engineering, University of Canterbury, Christchurch, New Zealand; 3 Department of Conservation, Biodiversity Group, Dunedin, New Zealand; University of New England, AUSTRALIA

## Abstract

Statistically robust monitoring of threatened populations is essential for effective conservation management because the population trend data that monitoring generates is often used to make decisions about when and how to take action. Despite representing the highest proportion of threatened animals globally, the development of best practice methods for monitoring populations of threatened insects is relatively uncommon. Traditionally, population trend data for the Nationally Endangered New Zealand grasshopper *Brachaspis robustus* has been determined by counting all adults and nymphs seen on a single ~1.5 km transect searched once annually. This method lacks spatial and temporal replication, both of which are essential to overcome detection errors in highly cryptic species like *B*. *robustus*. It also provides no information about changes in the grasshopper’s distribution throughout its range. Here, we design and test new population density and site occupancy monitoring protocols by comparing a) comprehensive plot and transect searches at one site and b) transect searches at two sites representing two different habitats (gravel road and natural riverbed) occupied by the species across its remaining range. Using power analyses, we determined a) the number of transects, b) the number of repeated visits and c) the grasshopper demographic to count to accurately detect long term change in relative population density. To inform a monitoring protocol design to track trends in grasshopper distribution, we estimated the probability of detecting an individual with respect to a) search area, b) weather and c) the grasshopper demographic counted at each of the two sites. Density estimates from plots and transects did not differ significantly. Population density monitoring was found to be most informative when large adult females present in early summer were used to index population size. To detect a significant change in relative density with power > 0.8 at the gravel road habitat, at least seventeen spatial replicates (transects) and four temporal replicates (visits) were required. Density estimates at the natural braided river site performed poorly and likely require a much higher survey effort. Detection of grasshopper presence was highest (*p*_*g*_ > 0.6) using a 100 m x 1 m transect at both sites in February under optimal (no cloud) conditions. At least three visits to a transect should be conducted per season for distribution monitoring. Monitoring protocols that inform the management of threatened species are crucial for better understanding and mitigation of the current global trends of insect decline. This study provides an exemplar of how appropriate monitoring protocols can be developed for threatened insect species.

## Introduction

Threatened species management routinely relies on population trend assessments to make decisions about when to invoke action [1, 2] and to measure the conservation benefit an action has provided [3]. Few insects benefit from scientifically developed and tested conservation monitoring protocols, despite 1,819 species currently (as of July 2020) classified as threatened in the IUCN red list assessments, and thousands more recognised as threatened under country specific assessment criteria such as the New Zealand Threat Classification system [4]. Common tools for monitoring insect diversity or pest species such as pitfall, malaise and sticky traps [5–8] are often unsuitable for threatened species because they are lethal and targeted to abundant and mobile species. A rich body of literature explores and develops the use of Pollard walks as a monitoring protocol for threatened Lepidoptera [9, 10], but the protocols are not transferable to many other insects, especially those that are elusive or cryptic. Mark-recapture has been used to monitor a range of threatened insects [11] including beetles [12], butterflies [13, 14] and grasshoppers [15–17]. In addition to drawbacks like high time investments to capture, handle and mark individuals, mark-recapture can be problematic for species with long juvenile phases, short adult life spans and small adult populations because marks are lost between moults, the time available to study adults is limited, and recaptures can fall below the threshold needed to produced valid estimates. To fully understand the global trends in insect species loss [18–20], a broader suite of monitoring methods needs to be developed that can be adapted for species of concern and used to underpin effective management.

The New Zealand insect fauna comprises of ~20,000 species, of which ~10% have been assessed under the New Zealand Threat Classification System (NZTCS), a National ranking system analogous to the IUCN red list [4]. Of these, 849 are listed as Threatened or At Risk. The robust grasshopper, *Brachaspis robustus*, is a flightless New Zealand endemic restricted to the Mackenzie Basin, and is classified as Threatened: Nationally Endangered [21, 22]. It prefers open, stony habitat, and primarily inhabits the wide dynamic riverbeds and rocky terraces of braided rivers. The current accepted distribution of *B*. *robustus* is based on maps created during bird surveys and other work more than 25 years ago [23] supplemented with irregular partial surveys at a limited number of sites, and *ad hoc* observations since. Most riverbed habitat that is known to be occupied by *B*. *robustus* has been modified to varying degrees by hydro-electric dams and introduced species including weedy vegetation and predatory mammals [24]. The most well studied, and one of the densest populations of *B*. *robustus* occupies an un-used gravel road [25]. The grasshopper is a non-stridulating species with colouration that strongly mimics the substrate of its habitat. It is extremely cryptic and detection of an individual (including the large adult females that reach up to 36 mm in length) usually requires the grasshopper to move. Detection is most common when an individual jumps in response to observer approach, but is compromised if the individual responds by taking refuge underneath rocks or other habitat features [26], or does not jump because of cold and/or overcast weather. The highly cryptic nature of *B*. *robustus* leads to significant detection errors during population monitoring [27].

Recent attempts at annual population monitoring of *B*. *robustus* have focused on the population that is restricted to a narrow unused gravel road. Monitoring within this habitat has consisted of a single transect searched annually by two observers on a single day in February during warm, sunny weather (Te Manahuna/Twizel Department of Conservation, pers. comms.). One-off annual surveys of *B*. *robustus* are known to produce highly variable counts, and are unlikely to provide accurate population data [28]. Furthermore, the *B*. *robustus* population present in February is mostly comprised of nymphs [29], which have been shown to have significantly lower individual detection probabilities than the larger adults of the species [27]. It has been suggested that adult female grasshoppers should be used as an indicator of population density to maximise both biological relevance and detection [27].

Regular monitoring at the landscape scale that tracks abundance and distribution of *B*. *robustus* across its full range would also inform better conservation management of this species. Although the species is known to be patchy within its range [26, 28], the drivers of patchiness have not been fully resolved. Additionally, the reported distribution of *B*. *robustus* may not reflect the true current distribution of the species because poor detectability and a lack of targeted monitoring may have resulted in false absences throughout the landscape, or conversely, failure to detect continued range contraction. Occupancy modelling may provide a suitable monitoring method that can be used to both model *B*. *robustus* distribution and to monitor the changes in distribution over time with respect to habitat degradation or conservation management action [30]. A particular benefit of this method is that it incorporates detection histories and accounts for false absences during monitoring [31].

In the current study, we use *B*. *robustus* as an example species to demonstrate how monitoring protocols can be developed for cryptic threatened insects to improve data quality and maximise conservation outcomes. Our objectives are to determine; 1) is there a difference in population estimates generated using a transect search method compared to a plot search method; 2) what is the optimal monitoring design required to detect a significant change in population density over time in the two habitat types occupied by *B*. *robustus* with respect to a) the number of transects, b) the number of repeated visits and c) the grasshopper demographic counted; 3) how do we maximise the probability of grasshopper detection for distribution monitoring with respect to a) search area, b) the number of repeated visits and c) the grasshopper demographic counted. We are confident that the principles used to design appropriate monitoring protocols for *B*. *robustus* can be applied to the development of monitoring protocols for other threatened and/or cryptic insects.

## Materials and methods

*Brachaspis robustus* is endemic to very specific areas within a ~7,339 km^2^ intermontane basin in the centre of New Zealand’s South Island. The grasshopper is a braided river specialist that occupies open rocky habitats associated with river floodplains and terraces [26, 32]. It has an estimated life span of approximately 28 months from the time the egg is laid to the end of the resulting adult’s life [29]. Eggs are laid during the summer, and nymphs hatch the following summer. Eggs likely require winter conditions to complete development, thus preventing hatching in the same season they are laid. Juveniles overwinter then reach adulthood the following summer, then they reproduce and likely die before the onset of winter. Therefore, two to three generations at different stages of development co-occur throughout the year [27].

Over the past c. 200 years, braided river habitats in the basin have become increasingly impacted by human settlement and development. The development of a basin-wide hydroelectric scheme has reduced natural flows and disrupted flood dynamics in many of the rivers, making the associated riverbeds vulnerable to invasion by exotic weedy species [24, 33] that reduce the availability of open rocky habitats required by *B*. *robustus* for basking and oviposition. Weedy vegetation also provides habitat for introduced predatory mammals [34] including mustelids, cats (*Felis catus*) and hedgehogs (*Erinaceus europaeus*) that will prey opportunistically upon *B*. *robustus* [26, 35].

### Site descriptions

**Fig 1:**
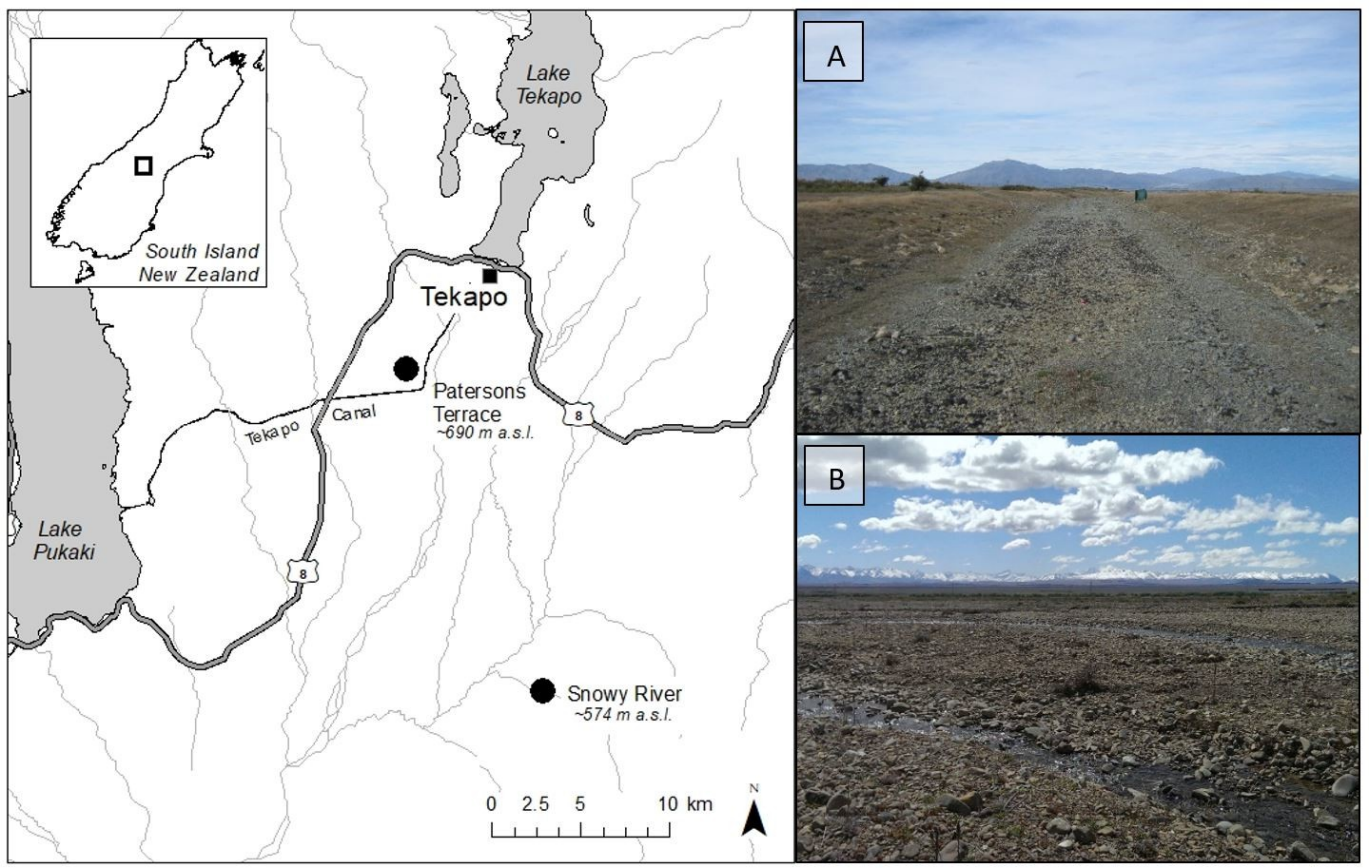
The location of Patersons Terrace and Snowy River in the Mackenzie basin in the central South Island, New Zealand (inset). (A) Patersons Terrace, an unused gravel road. (B) Snowy River, an alluvial fan with braided river characteristics. This figure contains data sourced from the LINZ Data Service licensed for reuse under CC BY 4.0.

This study was conducted at two sites in New Zealand. The first, Patersons Terrace (Fig 1A), is an unused gravel road situated ~8 km SW of Tekapō. It is not known when or how *B*. *robustus* colonised this site. The road substrate is comprised of gravels (small stones < 64 mm Ø) and larger cobbles that have been compacted by historical heavy vehicle use. Vegetation is sparse and consists mostly of lichens and low stature vascular plants such as *Raoulia australis* and hawkweed (*Hieracium* spp. and *Pilosella* spp.), as well as occasional *Rosa rubiginosa* and exotic grasses. The gravel habitat is bordered by semi-modified grasslands dominated by fescue tussock (*Festuca novae-zelandiae*) and exotic pasture grass [36]. Permission to access Patersons Terrace was granted by Genesis Energy and Land Information New Zealand (LINZ).

The second site, Snowy River (Fig 1B), is an alluvial fan with braided river characteristics which flows intermittently throughout the year. *Brachaspis robustus* are established on the lower braided section (~ 600 m wide). Occasional flooding events disturb the riverbed and remove vegetation. Mosses, lichens and herbaceous plants (e.g., introduced Viper’s bugloss, *Echium vulgare*), establish quickly after flooding events, and larger woody plants (e.g., *R*. *rubiginosa*) are present on more stable sections of the riverbed. Substrate size is diverse and includes fine sands, cobbles and large boulders. Permission to access Snowy River was granted by Grampians Station.

### Field methods

Plot and transect searches of equal area were compared at Patersons Terrace during the austral summer (November to March) for three consecutive seasons (2015–2018). Three 100 m x 1 m (100 m^2^) transects, spaced ~1 km apart, were marked out along the centre of the gravel road. Along each 100 m transect, four evenly spaced 5 m x 5 m (25 m^2^) plots were defined on alternating edges of the road using metal corner pegs. Henceforth, a group of 4 plots (equal to 100 m^2^) is referred to as a “plot unit”. The plots and transects remained in the same location over the duration of the study.

Transect searches were conducted at Snowy River during the second (2016–17) and third (2017–18) seasons only. Three 100 m x 1 m transects spaced > 200 m apart were set up longitudinally along the riverbed in locations where grasshoppers were known to occur. Transects were marked with plastic pegs placed at 20 m intervals. In the third season, two additional 100 m x 1 m transects were set up at the site. The three original transects were set up as close as possible to their initial location. However, changes in channel morphology resulted in minor deviations of less than 8 m.

Monitoring took place from November to March each summer on days of suitable weather (ground temperature > 13.6°C and not during gale force winds or precipitation). Each transect and plot unit was searched on at least 6 days per month when feasible (Patersons Terrace, mean visits per month = 6, range = 2 to 8; Snowy River, mean = 5.5, range = 3 to 7) but the number of monthly searches achieved and duration between visits (1 to 11 days) varied according to the occurrence of favourable weather. All plots and transects were searched by the same observer throughout the study. Both sites were searched on the same day except where weather was not permitting (e.g., rain at one of the sites). Prior to commencing a search, barometric pressure, air temperature at 1 m above the ground, and ground surface temperature in the shade were measured using a Kestrel 3500 Pocket Weather Metre (GeoSystems New Zealand Ltd). Cloud cover (categories: none; high cloud, when cloud was high in the sky but did not cause shadows; patchy cloud, when clouds were lower in the sky and caused shadows; overcast) and start time were recorded for each search. During the search, the observer walked slowly sweeping their front foot over the ground in front of them and moved in a direction such that their shadow fell on the area already searched. This method made visual detection possible by eliciting a jump response from the grasshopper. When detected, grasshoppers were captured, their body length (from the top of the head to the tip of the abdomen) and femur length measured, and sex and transect location recorded. Each grasshopper was then released behind the observer to ensure it was not re-counted, and the remainder of the area was searched. The minimum time required to complete a search of a 100 m^2^ transect was 5 mins, and for each plot was ~1.5 mins (~6 mins per 100 m^2^ plot unit including time taken to walk between plots) but this increased with the number of grasshoppers found. During this study, adulthood was determined by measuring hind femur length. Females with a femur length of ≥ 15 mm, and males with a femur length of ≥ 9 mm were considered adults. Individuals less than 8 mm in body length were excluded from the study because the risk of causing a fatal injury during capture and handling was too high. Permission to handle a Threatened species for the purpose of this research was provided by the Department of Conservation, New Zealand (DOCDM-1528162).

### Statistical analyses

#### Search method comparisons (transect versus plot)

All analyses were conducted in *R* [37] unless otherwise stated. For all three seasons of sampling at Patersons Terrace, total grasshopper counts from transect searches (three transects of 100 m x 1 m) and plot unit searches (three plot units comprised of four 5 m x 5 m plots) were pooled to give a count per day for each search method over a combined 300 m^2^ search area. The daily count generated from the two search methods was compared using a generalised linear model. A negative binomial distribution was fitted using *MASS* [38] to account for overdispersion in the data. Model fit was checked by ensuring dispersion and Pearson’s χ^2^ was below the χ^2^ 5% critical value. Search method, month, and season were specified as covariates. Post-hoc pairwise comparisons of co-variates was conducted using *multcomp* [39] and a Tukey distribution. The mean daily count generated by the two methods was also compared for adult female *B*. *robustus* separately. Input data was limited to November and December counts to coincide with peak adult female abundance. Season, month and search method were specified as covariates in a Poisson model fit in *lme4* [40]. Model fit was checked as above. Post-hoc pairwise comparisons of co-variates was conducted using *multcomp* [39] and a Tukey distribution.

For seasons 2 and 3, the Index of Dispersion (D = σ^2^/μ) was used to calculate how much variability there was in the total population count generated by each sampling method at Patersons Terrace (plot units and transects) and Snowy River (transects only) within each month (November to March) and compared using a linear mixed effects model in *lme4* [40]. Search method (categories: Patersons Terrace transects, Patersons Terrace plots, Snowy River transects) was specified as a fixed effect, and season and sampling unit were specified as random effects. Residuals were visually assessed for normality and a slight left skew existed. Removal of outliers (D ≥ 4) improved normality of residuals but did not affect model output, therefore outliers were retained in the dataset analysed.

#### Detecting population density trends

Power analysis (using *R* version 3.5.0) was used to determine the number of transects and visits (survey replicates occurring on different days) that were required to detect a significant change in population size (p < 0.05) at Patersons Terrace and Snowy River with a power of 0.8. The analyses were conducted on four data subsets so that any new monitoring recommendation could be compared to the historic protocol at both habitats; adult female counts collected throughout November and December at Patersons Terrace (1), and Snowy River (2), and adult and nymph counts of both sexes collected in February at Patersons Terrace (3), and Snowy River (4). The count of grasshoppers detected on a transect during each visit was modelled using a generalised linear mixed effect model with a Poisson distribution in *lme4* [40]. Year was modelled as a fixed effect and the number of transects and visits were random effects. We considered the addition of a random effect parameter at the observation level to the model. The parameter was only retained in the model if it explained a significant (p < 0.05) amount of variation. If there was no significant difference (p ≥ 0.05) between the models when compared using ANOVA, then the model with the lowest AIC score was selected. Using the model parameters, count data was predicted 1000 times for each combination of 25 visits and 40 transects. If a simulation resulted in a computational error, it was assumed that no significant difference could be detected, and p was set to 1. The power to detect significant (at p < 0.05) change in population size was calculated by dividing the number of simulations producing a significant p-value by the total number of simulations run.

#### Maximising detection of species presence

To inform an occupancy modelling design for *B*. *robustus*, the probability of detecting 1) a grasshopper of any age or sex, and 2) an adult female grasshopper, on a 100 m x 1 m transect in an area known to be populated (compared to detecting zero grasshoppers), was estimated using a generalised linear mixed effects model with a binomial distribution in *lme4* [40]. Study site (Snowy River, Patersons Terrace), month (November, December, January, February, March), cloud cover (no cloud, high cloud, patchy cloud, overcast) and ground temperature were considered as fixed effects in the model, and season and transect were specified as random effects. Temperature did not have a significant effect and because of missing values, was excluded from further analyses and model selection. Model selection from nested models was conducted using ANOVA with a χ^2^ test and selecting for lowest AIC. Model fit was assessed by visually checking for overdispersion. The analysis was repeated using a 20 m x 1 m resolution. Each 100 m^2^ transect was divided into five 20 m^2^ sections, and presence or absence of grasshopper detection within each segment was determined from the locations that were recorded when each grasshopper was captured. Pairwise comparisons were conducted in *lsmeans* [41], using a Tukey adjustment, and visualised using *multcompView* [39].

## Results

### Search method comparisons (transect versus plot)

**Fig 2:**
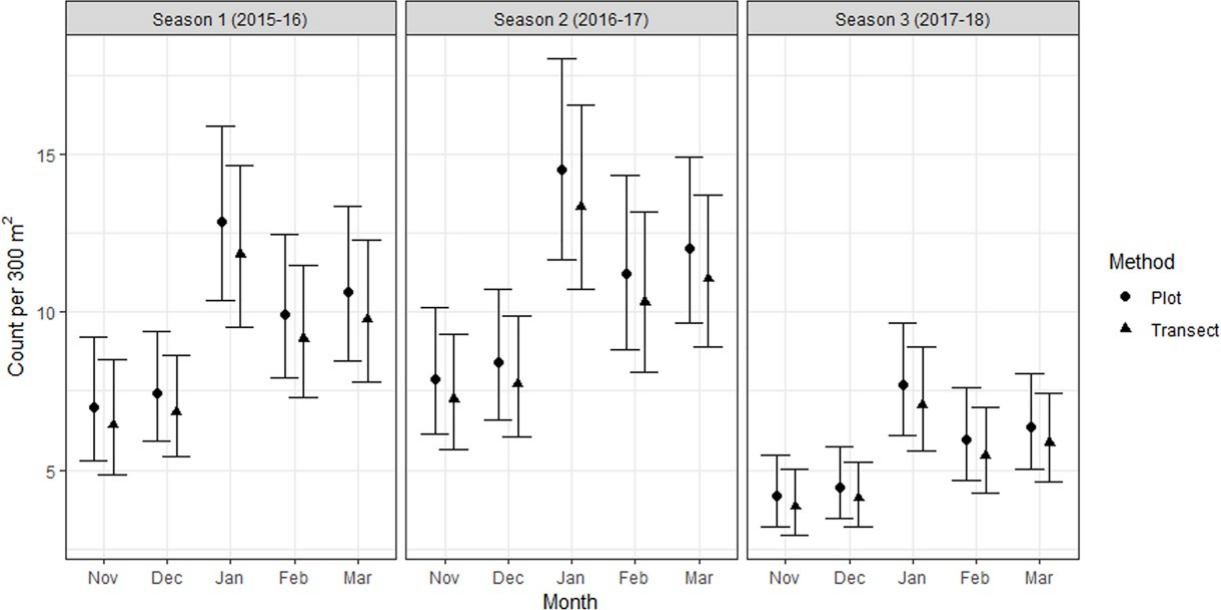
The predicted mean number (± SE) of *B*. *robustus* grasshoppers within the 300 m^2^ sampling area using plot searches (12 plots x 25 m^2^) and transect searches (3 transects x 100 m^2^) at Patersons Terrace during the monitoring period (November–March) for three seasons (2015–16 to 2017–18). Excludes grasshoppers less than 8 mm in body length.

The number of individuals counted using transects was on average 8% lower than counts from plots, but the difference was not significant (p = 0.32). On average, there were significantly more grasshoppers counted in January than December (p < 0.001) and November (p < 0.001), and in March than November (p = 0.03). No other pairwise comparisons between months were significant (i.e., p > 0.05). Season 1 and 2 counts were not significantly different (p = 0.46) but counts in season 3 were on average 41% lower than season 1 (p < 0.001), and 47% lower than season 2 (p < 0.001; Fig 2).

**Fig 3:**
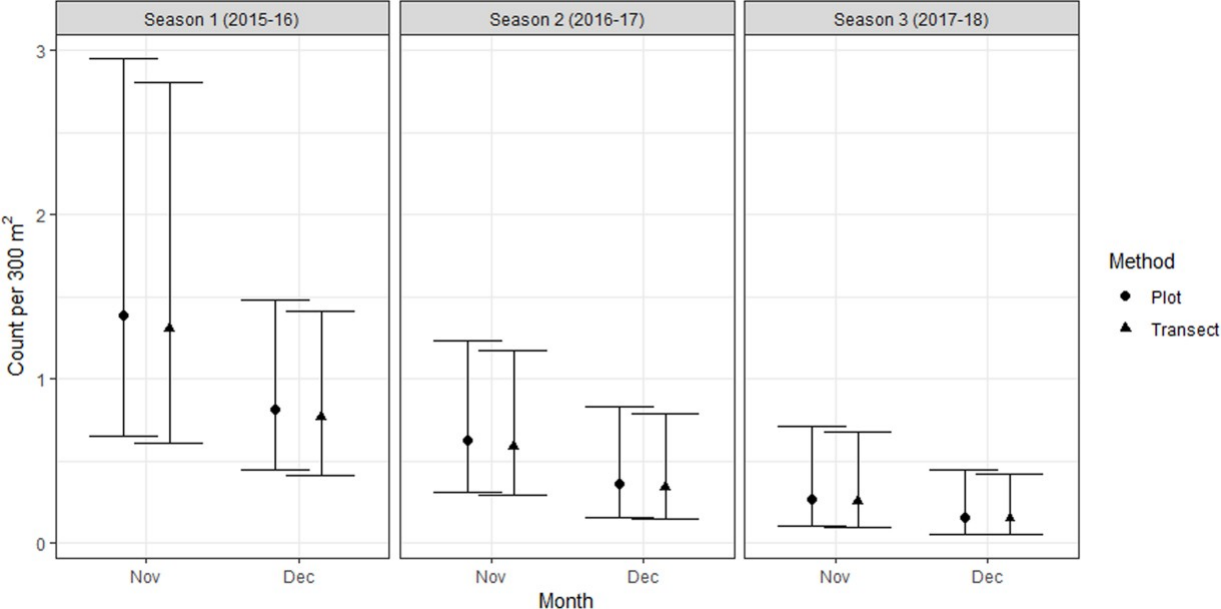
The mean number (± SE) of adult female *B*. *robustus* grasshoppers detected in 300 m^2^ of sampling area using plot searches (12 plots x 25 m^2^) and transect searches (3 transects x 100 m^2^) at Patersons Terrace during peak adult occurrence (November and December) for three seasons (2015–16 to 2017–18). Female grasshoppers with a femur length ≥15 mm were considered to be adult.

On average, searches using transects detected 6% fewer adult females than did plots, but the difference was non-significant (p = 0.87). Counts were not significantly different between November and December (p = 0.15). There were significantly fewer adult females detected on average in season 3 than season 1 (p = 0.004), but the differences between seasons 2 and 1 (p = 0.11), and 3 and 2 (p = 0.26) were non-significant (Fig 3). There was no significant difference in the Index of Dispersion for full population data collected using plot searches, or transect searches at either site (F_(2,92.2)_ = 1.71, p = 0.19).

### Detecting population density trends

**Fig 4:**
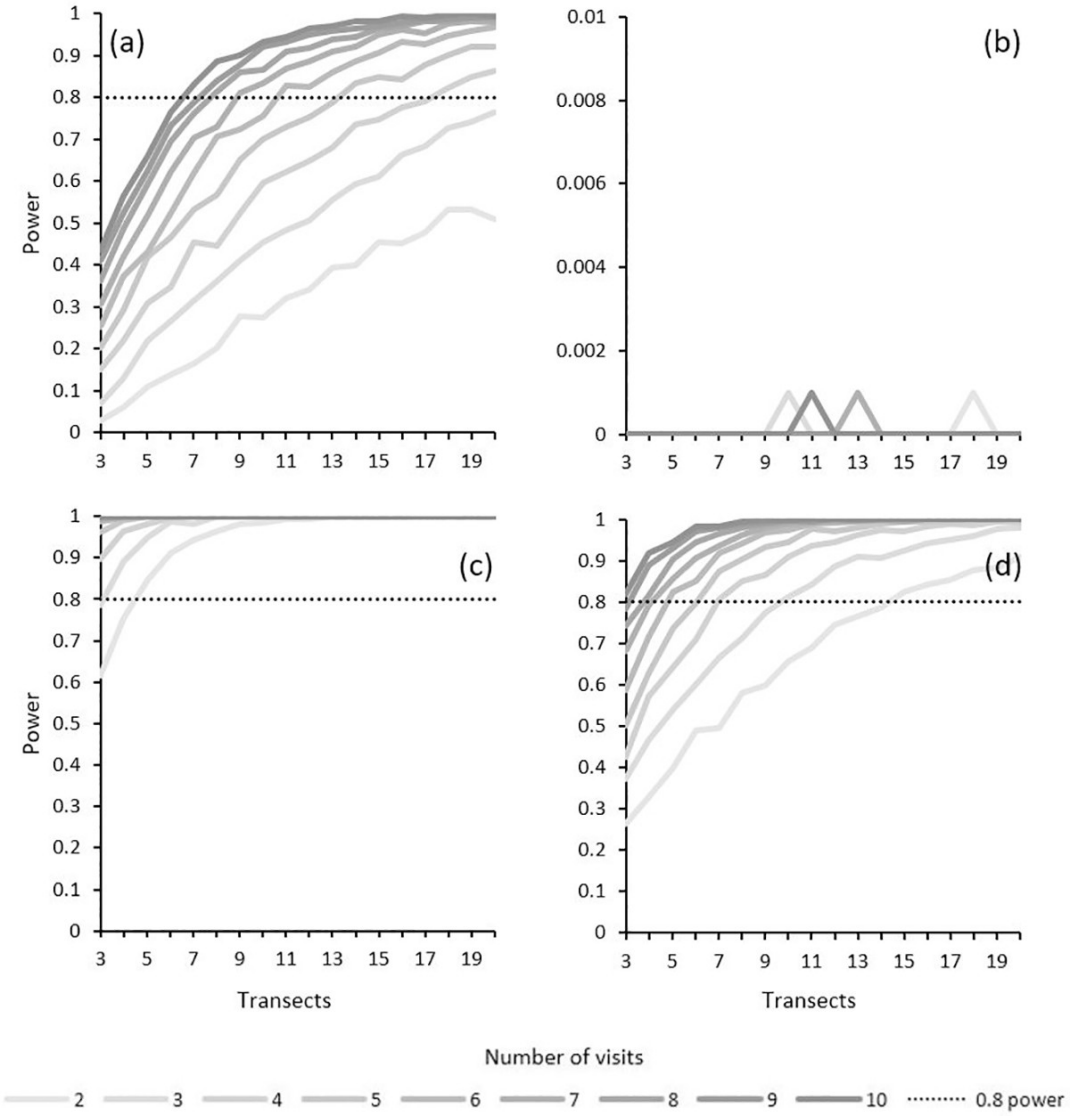
The power to detect a significant (p < 0.05) change in *B*. *robustus* population size with increasing number of transects and repeated visits using adult female data collected in November and December at (a) Patersons Terrace, and (b) Snowy River, and total population (any age or sex) data collected in February (as per historical monitoring methods for *B*. *robustus*) at (c) Patersons Terrace, and (d) Snowy River. Female grasshoppers with a femur length ≥15 mm were considered to be adult. Excludes grasshoppers less than 8 mm in body length.

At Patersons Terrace, we estimated the adult female grasshopper density in November to be 0.5 individuals per 100 m^2^ in season 1 (*n* = 2 temporal replicates), 0.14 (*n* = 7) in season 2, and 0.17 (*n* = 6) in season 3 at Patersons Terrace, and 0.44 (*n* = 3) in season 2 and 0.07 (*n* = 6) in season 3 at Snowy River. The power to detect a significant change (p < 0.05) in population size at Snowy River when monitoring adult females in November and December was low for all combinations of transect counts and repeated visits modelled. At Patersons Terrace, power of 0.8 could be achieved using a minimum of 7 transects and 10 repeated visits, or 17 transects and 4 repeated visits. Power was much higher for both sites when all demographics were included in counts in February (Fig 4).

### Maximising detection of species presence

**Fig 5:**
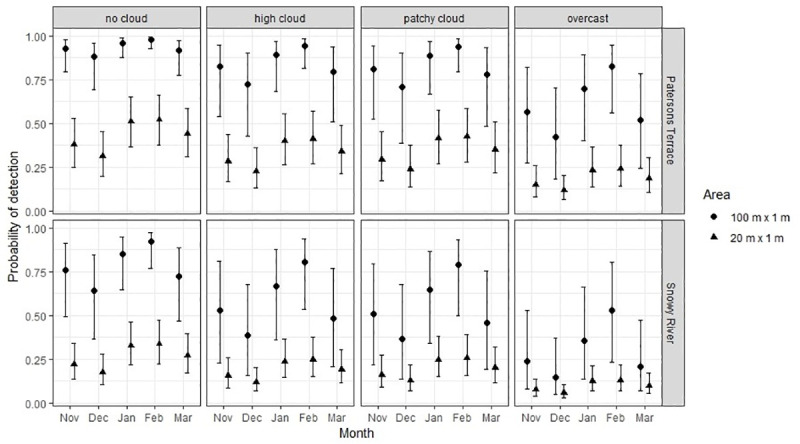
The probability (± SE) of detecting a *B*. *robustus* grasshopper of any demographic on a 100 m x 1 m (100 m^2^) and 20 m x 1 m (20 m^2^) transect at Patersons Terrace and Snowy River under four different cloud conditions: No cloud, high cloud, patchy cloud and overcast. Excludes grasshoppers less than 8 mm in body length.

**Fig 6:**
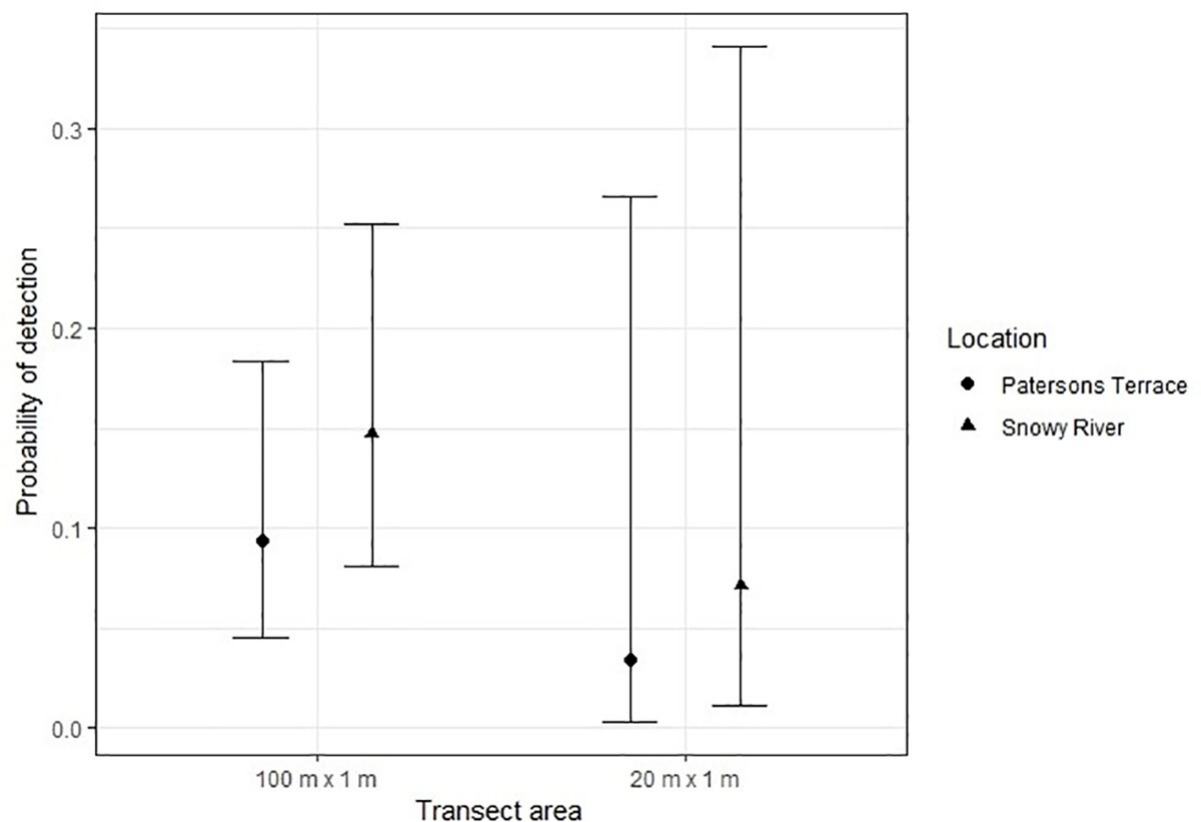
The probability (± SE) of detecting an adult female *B*. *robustus* along a 100 m x 1 m (100 m^2^) and 20 m x 1 m (20 m^2^) transect at Patersons Terrace and Snowy River in November or December. Female grasshoppers with a femur length ≥15 mm were considered to be adult.

Detection probabilities for distribution monitoring were generally lower using 20 m x 1 m transects (20 m^2^ search area) compared to 100 m x 1 m transects (100 m^2^ search area), but showed similar trends with respect to site, cloud cover and month (Fig 5). Holding month and cloud cover constant, the probability of detecting any grasshopper (*p*_*g*_) was on average lower at Snowy River than at Patersons Terrace (20 m^2^, 55% lower, p = 0.001; 100 m^2^, 53% lower, p = 0.003). The probability of detecting a grasshopper was highest and less variable under ‘no cloud’ conditions (20 m^2^, *p*_*g*_ = 0.32; 100 m^2^, *p*_*g*_ = 0.88) and was lowest when ‘overcast’ (20 m^2^, *p*_*g*_ = 0.12; 100 m^2^, *p*_*g*_ = 0.42), and was higher in January (20 m^2^, *p*_*g*_ = 0.40; 100 m^2^, *p*_*g*_ = 0.89) and February (20 m^2^, *p*_*g*_ = 0.41; 100 m^2^, *p*_*g*_ = 0.94) than for any other month (November, December, March). The probability of detecting an adult female *B*. *robustus* was less than 0.15 at both Patersons Terrace and Snowy River for both 100 m^2^ and 20 m^2^ transect lengths (Fig 6).

## Discussion

This study represents an intensive, multi-seasonal assessment of population monitoring for a highly threatened and elusive insect. It has provided key insights that are fundamental to informing the design of a monitoring protocol suitable for *B*. *robustus*, a flightless riverbed specialist. It also highlights several important considerations for the design of monitoring protocols for threatened insects more broadly. In particular, we were able to take into account statistical power, biological relevance and practical limitations when selecting the ideal protocols to meet the monitoring objectives for our focal species. By taking this approach, we have been able to develop recommendations for an appropriate search area shape, search frequency, timing within the season, weather conditions, and indices (all grasshoppers or only adult females) for population density and distribution monitoring.

Based on our experience with *B*. *robustus*, we recommend conservation practitioners working with similarly threatened and/or cryptic insects undertake a robust approach to optimise monitoring such that the data collected is accurate, replicable over time and space, and able to address the objectives of their programme without wasting effort. Early investment in determining an appropriate monitoring design has the potential to significantly improve the conservation management of individual species and will provide a valuable addition to the limited body of knowledge on monitoring threatened and cryptic insect species that is desperately needed to address the continued decline of insects globally.

### Search method comparisons (transect versus plot)

Grasshopper counts generated from plot searches and transect searches at Patersons Terrace did not differ significantly, although mean plot counts were slightly higher. We expected a greater difference given transects had 202 m of exposed edge per 100 m^2^ search area, compared to 80 m for plots, meaning opportunities for grasshoppers to enter and exit the search area during the monitoring event were much higher during transect searches. Additionally, during a plot search the observer’s direction of movement loops back and forth within the plot, meaning they spend more time within a grasshopper’s jump range. This provides more opportunities for accidental double-counting to occur and could have artificially inflated counts relative to the transect method where grasshoppers are released behind the observer. In this study, we believe the above effects were minimised by short search durations and the small area of the plots that allowed the observer to keep track of individual grasshoppers and avoid double-counting. However, when monitoring species with extremely low densities, double-counting could cause significant errors in trend data. For *B*. *robustus*, we found the mean density of adult females to range between 0.07 and 0.5 individuals per 100 m^2^. Double counting of even a few individuals could artificially inflate population density estimates to the point that a false trend might be generated. For threatened insects, falsely inflated population estimates and/or obscured population trends could have significant implications if they were to result in management delays or complete inaction when it is needed.

Conducting threatened species monitoring in locations where key resources of the target species occur is an important consideration. Retaining static transect locations for multiple years might be suitable for species that inhabit relatively homogenous environments, such as grasslands, or are associated with specific host plants with a stable distribution, but it could be problematic if the underlying resource distribution shifts and transect locations do not reflect that change [42]. For terrestrial braided river insects like *B*. *robustus*, one of the most significant changes in resource distribution will be associated with seasonal changes to channel morphology driven by flooding events. For example, the braided river grasshopper *Bryodema tuberculata* persists as metapopulations, where flooding or succession causes local extinctions that are compensated for by the recolonisation of new gravel bars [43]. Flexibility in search locations is vital to avoid mistaking a change in distribution of the species in the landscape for a sudden decline in population density.

When implementing monitoring over larger areas, it is likely that an observer will use a GPS to navigate rather than static markers as used here. A transect search only requires navigation between two points. A plot search, in contrast, would require navigation back and forth multiple times without crossing the same path, which is much more difficult to achieve (pers. obs.) and has a higher risk of double-counting individual insects. Given little difference in counts or search time were found between plot and transect search methods in this study, a transect method is therefore recommended for monitoring *B*. *robustus* population density to reduce the risk of artificially inflated counts.

### Detecting population density trends

The importance of temporal replication during insect monitoring (i.e., multiple visits within a single season) was highlighted by several of the density estimates that we presented here for *B*. *robustus*. Density estimates for adult females in November at Patersons Terrace were similar in 2016 and 2017 when searches were replicated ≥ 6 times. When only 2 replicates were performed in 2015, the estimate was ~3 times higher. Similarly, at Snowy River, density estimates from adult females were ~6 times higher when only 3 visits were made compared to when 6 visits were made, and the same was true when adults and nymphs of both sexes were counted. Although an underlying annual effect on insect population size is to be expected (given the seasonal variation in temperature and other climatic influences), discrepancies of this magnitude are most likely to arise from detection variability, which is more prevalent when fewer temporal replications (visits) are undertaken [44].

Species phenology is a crucial consideration when making decisions about the design of threatened insects monitoring, particularly when the species is hemimetabolous and both nymphs and adults are present at the same time and difficult to distinguish (as is the case for *B*. *robustus*). We found that the power to detect significant change in population size was high when monitoring was conducted in February, even when there were as few as 5 transects visited 3 times. However, in February most of the *B*. *robustus* population is comprised of nymphs that are small in body size, have low probabilities of visual detection [27] and may be difficult for inexperienced observers to distinguish from other species [28]. Furthermore, grasshopper nymphs, and the juvenile stages of insects in general, typically have high rates of mortality [45], and for most threatened insects the relationship between nymph and adult numbers, and therefore recruitment, is unlikely to be known. We recommend that population density monitoring for *B*. *robustus* should index population size based on the relative density of large female grasshoppers, because they have the highest rates of visual detection and are representative of the breeding population [27]. Despite the fact that counts of large females were low, we found that monitoring using 20 transects (spatial replicates) and 4 visits (temporal replicates) per season provided sufficient power (> 0.8) to detect a significant (p < 0.05) change in population size at Patersons Terrace. Monitoring protocols reported for other threatened insects are also often restricted to the adult demographic, but most published examples are less complex because the species are either holometabolous (e.g., butterflies [9], beetles [46, 47]) or have aquatic nymphs occupying a different habitat (e.g., damsel- and dragonflies [48]) so that juveniles and adults cannot be confused; or they are hemimetabolous insects that are univoltine with synchronised development, or obviously winged (e.g., Crau Plain grasshopper [49]) and again adults and juveniles are unlikely to be confused.

In this study we demonstrated the development of a practical density monitoring protocol for the population of *B*. *robustus* present at Patersons Terrace, an un-used gravel road habitat. Our findings were not transferable to the population present at Snowy River, a natural braided river habitat. We found that the power to detect change in density of the large female demographic at Snowy River was very low, likely because our simulation was based on only 2 seasons of data that contained a high proportion of 0 counts. These low counts could indicate the population density is lower at Snowy River, but more likely they reflect poorer detectability in that environment. A review of methods for monitoring common grasshopper species in grasslands found that transect searches were accurate when the species occurred at low densities of less than 2 adults per m^2^ and in low open swards (versus higher densities and/or taller swards) provided environments were homogenous and movements into and out of the search area during the search period were minimal [50]. Patersons Terrace conforms to these requirements by design, as the substrate is uniform and compacted from historic vehicle use (i.e., relatively homogenous), and the habitat is narrow minimising the number of grasshoppers present on either side of a transect (i.e., low migration to/from search area). In contrast, Snowy River is a relatively expansive, active riverbed that provides a dynamic, heterogenous and refuge-rich habitat. These fundamental differences in habitat structure are likely to produce substantially different detection rates of large females during monitoring events.

Understanding the difference in the power to detect population density change between the two sites monitored here is crucial to the design of monitoring protocols for *B*. *robustus*, because the few remaining populations of the species can be broadly categorised as occupying two physically different habitats. Those that occupy relatively homogenous modified habitats, including Patersons Terrace, are easier to study and protect. The remaining populations occupy more natural riverbed habitats. These are more difficult to manage but are crucial to the persistence of the species in its natural range. To date, few targeted surveys have been undertaken for the riverbed populations and as a result there is little information on the grasshopper’s true current distribution. However, observations during regular management of other riverbed species suggest population contraction and increased distances between patches on the larger rivers. The data presented here indicate that monitoring to inform management of riverbed populations will likely need to be much more intensive than what is required at Patersons Terrace. Given resources for insect conservation globally are often extremely limited, practitioners will benefit from being flexible in the effort directed to monitoring at different sites to meet their conservation objectives, rather than taking a ‘one size fits all’ approach.

### Maximising detection of species presence

In general, when the probability of detection is high, fewer visits to a site are required to have confidence in the presence or absence of a species [51]. We found that the probability of detecting a grasshopper (*p*_*g*_) was higher at Patersons Terrace than Snowy River using either a 100 m or 20 m long transect. However, we could not tease apart whether this was a population density or habitat structure effect. If grasshopper density was higher, we would expect the probability of detection to be higher. This logic explains why *p*_*g*_ is higher in the months following nymph emergence, despite small juveniles having lower individual detection probabilities [27]. Alternatively, as mentioned above, *p*_*g*_ could also be higher at Patersons Terrace because the substrate is comprised of small, uniform and highly compacted gravels, and vegetation stature is low, so there are fewer refuges for grasshoppers to retreat into when disturbed by an observer. In contrast, Snowy River has diverse substrate size and seasonal riverbed disturbance creating interstitial spaces, as well as a higher diversity of vegetation, including both tall woody weeds and low leafy herbaceous plants that could conceal grasshoppers much more than the compact mat plants found at Patersons Terrace.

Conducting monitoring when probabilities of detection are high can provide greater confidence in monitoring results [10], particularly when the number of search replicates conducted is limited. One way to maximise detection probabilities is to restrict searches to when weather conditions are most favourable for detection [52]. In this study we found that cloud cover had a significant effect on the probability of detection. Because a jump in response to observer disturbance is usually required for detection to occur, monitoring that takes place on fine days will yield higher detection probabilities as grasshoppers are able to bask and raise body temperature, therefore increasing activity [53]. Although temperature was not found to be a significant predictor of detection probabilities in this study, it is usually a vital parameter for monitoring other insects, such as butterflies [52], and in this case might indicate that all our monitoring was conducted within the thermal thresholds for normal activity.

Mackenzie and Royle [51] recommend that a minimum of 3 visits be made when *p*_*g*_ > 0.5. Low *p*_*g*_ was yielded in both sites when using a 20 m^2^ transect, but a high *p*_*g*_ (> 0.6) was achieved at both sites using a 100 m^2^ transect under ‘no cloud’ conditions. However, the Patersons Terrace and Snowy River populations of *B*. *robustus* are expected to be the densest [25], and largest [26], across the species range respectively, so *p*_*g*_ is expected to be lower at other sites. We recommend that to monitor *B*. *robustus* distribution at a landscape scale, a minimum of 3 visits be made per season to transects ≥ 100 m in length during fine warm weather, and that visits occur in February when probabilities of detecting a grasshopper peak.

## Conclusions

An important finding from this research was that the best time to monitor the cryptic grasshopper, *B*. *robustus*, was dependant on whether the objective was to estimate population density or to determine population distribution. For *B*. *robustus*, *individual* detection probabilities peak in November and December when large female grasshoppers, the most visually detectable demographic, occur at the highest density [27]. Because this demographic is also the most biologically informative, and trends in relative abundance can be measured in homogenous habitats using the monitoring regime described above, November and December are the most appropriate time of year for measuring population *density* indices. However, the *species* detection probability peaks in February following nymph emergence, making this the best time of year for population *distribution* (site occupancy) monitoring to occur. This is an important contrast to monitoring of holometabolous insects where the appropriate timing within the season for both density and distribution monitoring is often the same, for example only adults of threatened butterflies may be monitored during peak flight period [10, 54].

Investing time into developing monitoring protocols that effectively measure changes in population density or distribution over time, as demonstrated here, directly benefit the conservation management of cryptic, threatened insect species. These benefits include ensuring conservation practitioners are best informed about current population size, trend and distribution when making critical management decisions, and providing a tool to measure the success of actions implemented to improve conservation outcomes. Although based on the observation of a single species, studies like this will collectively provide the methodology needed to obtain data that underpins our understanding of insect decline globally, and provide the basis to test the actions necessary to stabilise and reverse this decline, for the benefit of entire ecosystems.
